# Development of Global Chemical Profiling for Quality Assessment of *Ganoderma* Species by ChemPattern Software

**DOI:** 10.1155/2018/1675721

**Published:** 2018-03-04

**Authors:** Hui Zhang, Huijie Jiang, Xiaojing Zhang, Shengqiang Tong, Jizhong Yan

**Affiliations:** College of Pharmaceutical Science, Zhejiang University of Technology, No. 18 Chaowang Road, Hangzhou 310014, China

## Abstract

Triterpenoids are the major secondary metabolites and active substances in *Ganoderma*, considered as the “marker compounds” for the chemical evaluation or standardization of *Ganoderma*. A response surface methodology was used to optimize the ultrasonic-assisted extraction of triterpenoids. The extraction rate was 7.338 ± 0.150 mg/g under the optimum conditions: 87% ethanol, ratio of solid to liquid (w : v) 1 : 28, and ultrasound extraction time 36 min. Based on the high sensitivity and selectivity of HPLC-LTQ-Orbitrap-MS^*n*^, 24 components of triterpenoids were tentatively identified in the negative mode. Then, the global chemical profiling consisting of HPLC and TLC fingerprints generated by ChemPattern™ software was developed for evaluation of *Ganoderma* species. For fingerprint analysis, 11 peaks of triterpenoids were selected as the characteristic peaks to evaluate the similarities of different samples. The correlation coefficients of similarity were greater than 0.830. The cluster analysis showed a clear separation of three groups, and 11 peaks played key roles in differentiating these samples. The developed global chemical profiling method could be applied for rapid evaluation, quality control, and authenticity identification of *Ganoderma* and other herbal medicines.

## 1. Introduction

*Ganoderma*, a popular edible and medicinal mushroom, is commonly used as dietary supplements and touted as a remedy to promote health and longevity [1]. So far, more than 200 species of *Ganoderma* have been found in the world, and *Ganoderma lucidum* (Leyss. ex Fr.) Karst. and *Ganoderma sinense* Zhao, Xu et Zhang are officially recorded in Chinese pharmacopoeia [2]. Previous studies have demonstrated that *Ganoderma* possesses various biological properties, such as antitumor, antiaging, antioxidant, hyperglycemic, and regulating immunity [3–5]. Owing to its satisfactory clinical effects, more and more *Ganoderma* products as health foods or medicines have appeared at the market. However, it is hard to say whether its quality is good or bad, and it is also difficult to identify whether the raw materials are authentic or adulterant.

For *Ganoderma*, triterpenoids are the major secondary metabolites and active substances [6]. The pioneering has isolated and identified more than 300 triterpenoids from the spores, fruiting bodies, and cultured mycelia of *Ganoderma* [7–9]. Triterpenoids could be considered as the “marker compounds” for the chemical evaluation or standardization of *Ganoderma*. Professor Guo and his team's researches concluded that the content and composition of triterpenoids vary significantly due to difference in the strain, geographic origin, cultivation method, extraction process, and other factors [10, 11]. Besides, scholars have made a lot of exploratory work on the chromatographic fingerprint of *Ganoderma* products [12, 13]. However, the traditional fingerprints were always processed by the fingerprint similarity software (2004 or 2012 version), only limited to HPLC profiles without TLC. After that, researchers always apply other statistical software (such as SPSS and SAS) to process the data for cluster analysis or principal component analysis, which is relatively cumbersome and time-consuming. Therefore, it is essential to develop a global chemical profiling method for rapidly evaluating the quality of *Ganoderma* to ensure the efficacy.

Chemical profile reflects the totality of intrinsic chemical compounds of herbal medicines and emphasizes the integral characterization of a complex system [14, 15]. Here, the global chemical profiling method contained the establishment of HPLC and TLC fingerprints, the characterization of common peaks and statistical analysis. For processing the large scale of physical properties characteristic in the complex herbal extract, an advanced chemometric and chemical fingerprinting software was developed by Chemmind Technology Co., Ltd. ChemPattern is an advanced chemometric and chemical fingerprinting software, which endeavors to provide solutions for qualitative and quantitative quality evaluation and characteristic analysis. The software was employed to calculate the correlation coefficients between different chromatographic profiles, as well as to generate the representative standard fingerprint by mean simulation.

In the present work, triterpenoids were extracted from different *Ganoderma* samples under the ultrasonic-assisted condition optimized by response surface methodology (RSM) with the Box-Behnken design (BBD) [16]. The extract was analyzed by high-performance liquid chromatography coupled with linear ion trap-Orbitrap mass spectrometry (HPLC-LTQ-Orbitrap-MS^*n*^) for a comprehensive study of the multiple chemical constituents. In addition, the chromatographic data of HPLC and TLC were submitted into the professional ChemPattern software for establishing global chemical profiling and evaluating similarity. Furthermore, *Ganoderma* samples from different regions could be distinguished by clustering analysis and principal component analysis of fingerprint data.

## 2. Experimental

### 2.1. Materials and Reagents

**Figure 1:**
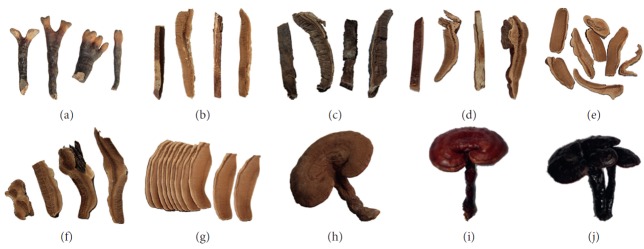
Different origins of *Ganoderma*: (a) Anhui: (b) Anhui: (c) America: (d) Anhui: (e) Jilin: (f) Anhui: (g) Anhui: (h) Henan: (i) Anhui: (j) Guangxi.

*Ganoderma* collected from different regions is shown in Figure 1. Samples were pulverized into powder and kept in a vacuum dryer. The standard sample of *Ganoderma* was identified by National Institutes for Food and Drug Control. Cellulase and oleanolic acid were obtained from Aladdin. Ganoderic acid A was purchased from Chengdu Must Bio-technology Co., Ltd. Acetonitrile and methanol were of HPLC grade and obtained from Anhui Tedia High Purity Solvents Co., Ltd. Ethanol, vanillin, sulfuric acid, acetic acid, perchloric acid, phosphoric acid, formic acid, petroleum ether, ethyl acetate, and other chemicals were of analytical grade.

### 2.2. Heating Reflux Extraction

Sample powder (2.0 g) and 95% ethanol (30 mL) were extracted under reflux at 75°C for 30 min. Then, the extraction solution was filtered through a filter paper and evaporated to dryness at 60°C.

### 2.3. Ultrasonic Extraction of Total Triterpenoids

Sample powder (2.0 g) and 95% ethanol (30 mL) were placed in an ultrasonic bath (300 W) at 75°C for 30 min. The suspension was cooled to room temperature and filtered. The filtrate was vacuum-dried at 60°C.

### 2.4. Determination of the Total Content of Triterpenoids

The total content of triterpenoids was determined according to the method of Hou with some modifications [17]. The oleanolic acid (2.0 mg) was dissolved in methanol (10 mL) to produce a standard solution. 0 *μ*L, 100 *μ*L, 200 *μ*L, 300 *μ*L, 400 *μ*L, 500 *μ*L, and 600 *μ*L standard solutions were added into a test tube, respectively, and evaporated in a water bath. 5% vanillin-acetic acid reagent (400 *μ*L) and perchloric acid (1000 *μ*L) were added, and the tube was placed in a water bath for 30 min at 65°C. When the reaction solution was cooled, acetic acid (5 mL) was added. The absorbance was determined at 546 nm using a microplate reader (Infinite®200 Pro NanoQuant, Tecan, Switzerland). The sample (100 *μ*L) was determined following the abovementioned method. Then, the weight of triterpenoids was calculated according to the standard curve. The extraction yield of triterpenoids was calculated as follows: yield (mg/g, w/w) = weight of triterpenoids/weight of raw materials. All determinations were performed in triplicates. The standard curve was *y* = 223.2*x* + 4.473, *R* ^2^ = 0.9950. The oleanolic acid weight in 20–120 *μ*g range showed a good linear relationship.

### 2.5. Experimental Design of RSM for Ultrasonic Extraction

Three independent factors of ultrasonic extraction were investigated using the RSM of BBD, including liquid-solid ratio (A: 15, 20, and 35 mL/g), extraction time (B: 35, 45, and 60 min), and ethanol concentration (C: 75, 85, and 95%), as shown in Table S1. The three levels were designated as −1, 0, and +1 for low, intermediate, and high values.

In order to predict the conditions of ultrasound extraction, experimental data were analyzed using the software Design-Expert version 8.06 and explained using the following nonlinear computer-generated quadratic model [18]:

(1) $$\begin{array}{c} R=\beta_{0}+\sum_{i=1}^{k}\beta_{i}x_{i}+\sum_{i=1}^{k}\beta_{ii}x_{i}^{2}+\sum_{i=1}\sum_{j=i+1}\beta_{ij}x_{i}x_{j}+\varepsilon , \end{array}$$

where *β*_0_ is the constant coefficient, *β*_*i*_, *β*_*ii*_, and *β*_*ij*_ are the coefficients for the linear, quadratic, and interaction effect, *x*_*i*_ and *x*_*j*_ are the independent variables, and *ε* is the error.

The adequacy of the model was tested through analysis of variance (ANOVA). The coefficients of determination *R* ^2^ and adj *R* ^2^ expressed the quality of fit of the resultant polynomial model, and the statistical significance was checked by *F*-value and lack of fit [19].

### 2.6. LC-LTQ-Orbitrap-MS^n^ Conditions

For accurate mass measurements, an Agilent 1290 HPLC instrument was coupled with a LTQ Orbitrap Velos mass spectrometer (Thermo Scientific, Hemel Hempstead, UK) equipped with an ESI source. An Eclipse plus C_18_ (50 mm × 4.6 mm, 1.8 *μ*m) column was used for chromatographic separation. The column temperature was kept at 35°C. The mobile phase consisted of acetonitrile (A) and 0.03% phosphoric acid solution (B). The gradient elution was as follows: 75–68% B at 0–15 min, 68–60% B at 15–20 min, 60–40% B at 20–25 min, 40–0% B at 25–40 min, and 0% B at 40–125 min. The DAD was set at 254 nm. The injection volume was 10 *μ*L, and the flow rate was 0.6 mL/min.

The operation parameters of mass spectrometry were as follows: source voltage, 4.0 kV; sheath gas, 20 (arbitrary units); auxiliary gas, 12 (arbitrary units); sweep gas, 2 (arbitrary units); and capillary temperature, 350°C. Default values were used for most other acquisition parameters: Fourier transformation (FT) automatic gain control (AGC) target 5 × 10^5^ for the MS mode and 5 × 10^4^ for the MS^*n*^ mode. Perfusion samples were analyzed in the data-dependent scan mode at a resolving power of 60,000 at *m*/*z* 400. The most intense ions were selected, and parent ions were fragmented by high-energy C-trap dissociation (HCD) with a normalized collision energy of 45% and an activation time of 100 ms. The maximum injection time was set to 100 ms with two microscans for the MS mode and to 1000 ms with one microscan for the MS^*n*^ mode. The mass range was from *m*/*z* 100 to 1500. Each sample was analyzed both in negative and positive modes. Data were analyzed using Xcalibur software version 2.2 (Thermo Fisher Scientific).

### 2.7. HPLC Chromatographic Fingerprint Analysis Conditions

HPLC chromatographic fingerprint analysis was conducted on a liquid chromatography system (1260, Agilent, America) equipped with a quaternary solvent deliver system, an autosampler, and a DAD (Agilent Technologies). The mobile phase consisted of acetonitrile (A) and 0.03% phosphoric acid solution (B) using a gradient elution of 75–68% B at 0–40 min, 68–60% B at 40–60 min, 60–40% B at 80–120 min, 40–0% B at 80–120 min, and 0% B at 120–125 min. Chromatographic separation was carried out at an Agilent Zorbax Extend-C_18_ column (4.6 mm × 250 mm, 5 *μ*m) with a solvent flow rate of 1.0 mL/min at a temperature of 35°C. The wavelength was set at 254 nm. The injection volume was 10 *μ*L.

### 2.8. TLC Chromatographic Conditions

Ganoderic acid A solution and samples a to k (5 *μ*L) were spotted using a microinjector on a 20 × 20 cm silica gel plate (GF254, Qingdao, China). The silica gel was activated in an oven at 80°C for 30 min before use. The mobile phase consisting of petroleum ether : ethyl acetate : formic acid (1 : 1 : 0.02, v/v/v) was added into a twin-trough chamber and saturated for 10 min. The plate in the chamber was developed upward over a path of 15 cm and sprayed with 1% vanillin-sulfuric acid solution. The plate was placed in an oven at 80°C for 10 min until the color of the triterpenoid spots was distinct. The image of TLC was reverse-phase processed, and the information of TLC spots was turned into the gray curve by software.

### 2.9. Statistical Analysis

All experiments were performed at least in triplicate. The values were expressed as means ± standard deviation (SD).

## 3. Results and Discussion

### 3.1. Comparison of Ultrasonic-Assisted Extraction and Reflux Extraction

Comparing heating reflux extraction (6.404 mg/g) with ultrasonic-assisted extraction (6.869 mg/g), the extraction yield of triterpenoids was increased significantly by ultrasonic-assisted extraction in the same extraction time with simple operation. The ultrasonic wave produced a strong cavitation, mechanical crushing, and thermal effect, which dissolved the active ingredients into the solvent more adequately and saved more energy. So, the ultrasonic-assisted extraction was selected for further optimization.

### 3.2. RSM for Optimization of Ultrasonic-Assisted Extraction

**Figure 2:**
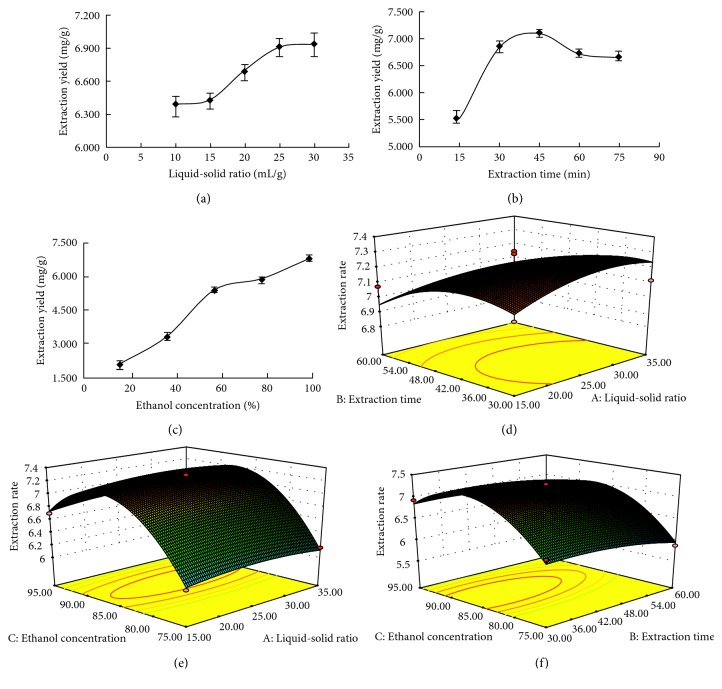
Effects of independent parameters: liquid-solid ratio (a), extraction time (b), and ethanol concentration (c) on the extraction yield of triterpenoids. Interaction effects between the liquid-solid ratio and extraction time (d), liquid-solid ratio and ethanol concentration (e), and extraction time and ethanol concentration (f) on the yield of triterpenoids.

The main factors which affected the ultrasonic extraction yield of triterpenoids were investigated by single-factor experiments, including the temperature, liquid-solid ratio, extraction time, and ethanol concentration. When the procedures were conducted at 30 min with a concentration of 95% ethanol, the maximum yield of triterpenoids was 0.6906% at the liquid-solid ratio 25 mL/g (Figure 2(a)). The liquid-solid ratio and concentration of ethanol were fixed at 25 mL/g and 95%. The result showed that the extraction efficiency increased to the maximum amount of 0.7097% at 45 min (Figure 2(b)). The yield of triterpenoids was significantly increased with the ethanol concentration varying from 15% to 95%, and the optimal ethanol concentration was from 75% to 95% (Figure 2(c)). Therefore, the liquid-solid ratio 25 mL/g, extraction time 45 min, and 85% ethanol were selected as the center points for each factor in the RSM experiments. The single-factor experiment of temperature showed that the extraction rate of triterpenoids was almost unchanged with increasing temperature after 50°C. So, the extraction temperature was not considered as the experimental factor of BBD and set at 50°C. An experimental program for optimizing the extraction of triterpenoids using RSM with BBD is shown in Table S2. The predicted values were obtained from the model fitting technique using the software Design-Expert version 8.06.

ANOVA was applied to optimize the extraction conditions of ultrasonic-assisted extraction for the triterpenoid yield and evaluate the relationship between response and variables. ANOVA for the response surface quadratic regression model showed that the *F*-value of model was 30.56 and the *P* value of model was smaller than 0.0001 (Table S3), suggesting the model was significant [16]. The coefficient of determination (*R* ^2^) of the model was 0.9752, and the adjusted determination coefficient (adj *R* ^2^) was 0.9433, which indicated good agreement between the experimental parameters and the predicted values of triterpenoids. The sequence of three factors influencing the triterpenoid yield was the ethanol concentration (C), extraction time (B), and liquid-solid ratio (A).

By statistically processing, the multiple second-order equation for the extraction yield of triterpenoids was obtained as follows:

(2) $$\begin{array}{c} Y=+7.23+0.015\text{A}-0.13\text{B}+0.32\text{C}-0.051\text{AB}+0.012\text{AC}+4.250\times 10^{-3}\text{BC}-0.075{\text{A}}^{2}-0.11{\text{B}}^{2}-0.72{\text{C}}^{2}, \end{array}$$

where *Y* is the extraction yield of triterpenoids, A, B, and C are the coded values of the liquid-solid ratio, extraction time, and ethanol concentration, respectively.

The extraction yield and the interaction of different variables could be predicted from the three-dimensional (3D) response surface (Figures 2(d)–2(f)). The steeper slope represented that the factor had a more significant effect on the extraction yield. The steeper slope of ethanol concentration implied that it had greater effect on the extraction yield of triterpenoids than on the extraction time and liquid-solid ratio.

According to the regression equation, the optimal parameters were the liquid-solid ratio 28.32 mL/g, extraction time 35.64 min, and ethanol concentration 87.25%. The theoretical highest yield of triterpenoids was 7.3118 mg/g predicted by the model. In order to validate the adequacy of the model, verification experiments were carried out by slightly modified conditions: liquid-solid ratio 28 mL/g, extraction time 36 min, and ethanol concentration 87%. The yield of triterpenoids of 7.338 ± 0.150 mg/g could be attained, which was 6.83% higher than the previous ultrasonic extraction method.

### 3.3. Characterization and Identification of Triterpenoids by LC-LTQ-Orbitrap-MS^n^

**Figure 3:**
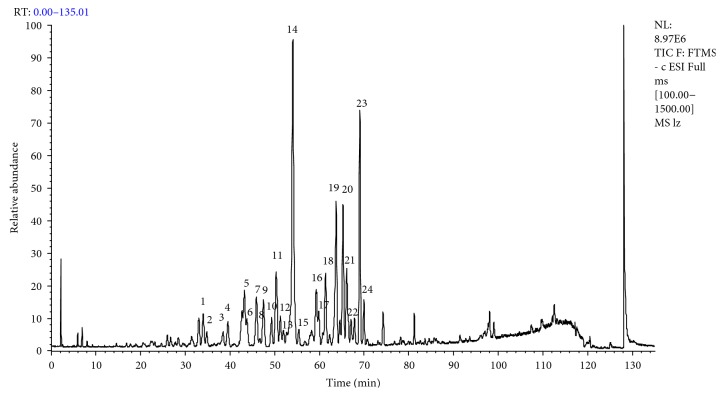
The total ion chromatograms (TICs) of the extract from *Ganoderma* by LC-LTQ-Orbitrap-MS^*n*^ in negative ion mode.

**Figure 4:**
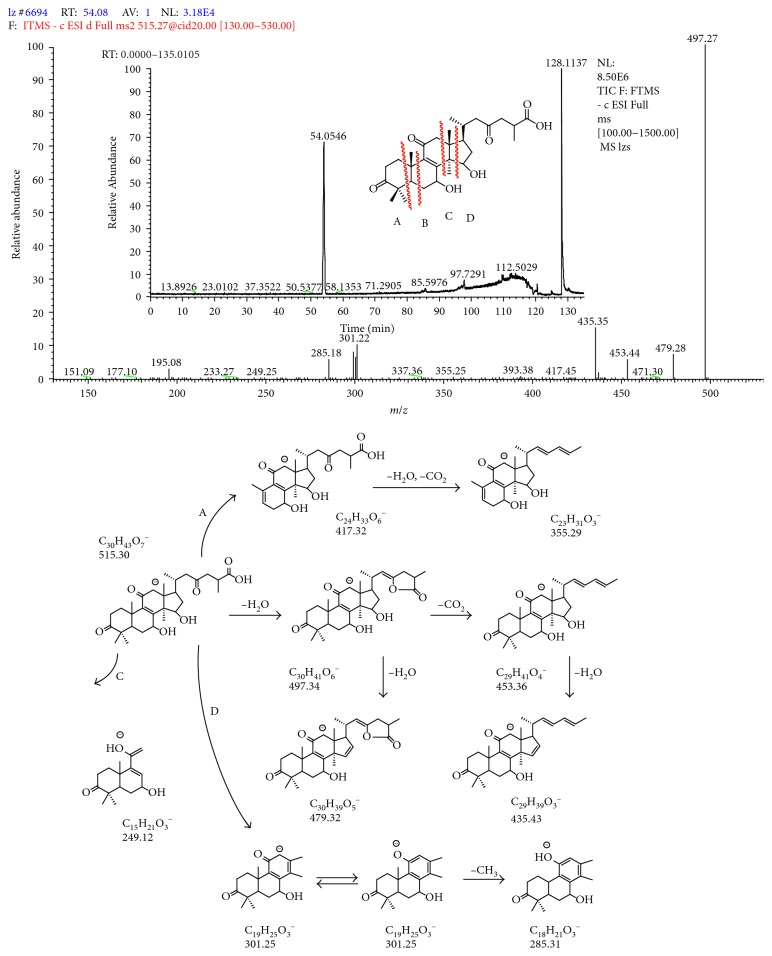
The mass spectrum and cleavage pathway of ganoderic acid A.

Qualitative analysis of triterpenoids was performed on the HPLC-LTQ-Orbitrap-MS^*n*^ system. ESI-MS spectra in both negative and positive modes were examined in this study. Negative-mode ESI was found to be sensitive for triterpenoids. All triterpenoids gave [M – H]^−^ ions in their negative ion mass spectra. The total ion chromatograms (TICs) of triterpenoids in the negative ion mode by LC-LTQ-Orbitrap-MS^*n*^ are shown in Figure 3. The fragmentation pathway of triterpenoids is summarized by using ganoderic acid A as the standard compound. The mass spectrum of ganoderic acid A and its major fragmentation pathways are given in Figure 4. In the negative ion mode, the prominent fragmentation pathways begin with the prominent losses of H_2_O or CO_2_; then, the cleavages took place on the A, C, and D rings. The [M – H]^−^ ion at *m*/*z* 515.30 (C_30_H_43_O_7_^−^) of ganoderic acid A produced a prominent ion at *m*/*z* 497.34 by eliminating a molecule of H_2_O (18 Da). The *m*/*z* 497.34 ion was further subjected to produce signals at m/*z* 479.32 or 453.36 by the sequential losses of H_2_O or CO_2_ (44 Da). The [M – H]^−^ ion at *m*/*z* 515.30 produced an ion at *m*/*z* 417.32 (C_24_H_33_O_6_^−^) by direct cleaving on ring A. The ion at *m*/*z* 355.29 could be obtained by the process of losing H_2_O and CO_2_, followed by cleavage of ring A. Ganoderic acid A was also cleaved on ring C to give the product ion at *m*/*z* 249.12 (C_15_H_21_O_3_^−^). The cleavage of ring D could be observed in ganoderic acid A, besides the cleavage of rings A and C. The [M – H]^−^ ion at *m*/*z* 515.30 produced an ion at *m*/*z* 301.25 (C_19_H_23_O_3_^−^). The *m*/*z* ion 301.25 then underwent losses of CH_3_ (15 Da) to generate an ion at *m*/*z* 285.31.

According to the nontarget compound identification strategy based on the accurate mass measurement (<5 ppm), MS/MS fragmentation patterns, diagnostic product ions, and different chromatographic behaviors, 24 compounds were unambiguously identified from triterpenoids [9, 10].  Table 1 summarizes the retention times (*t*_*R*_), molecular formula, [M – H]^−^ and CAS number of each compound, and MS/MS ions. The structures of 24 compounds are shown in Figure S1. These results provided the critical information for constructing chemical fingerprints of triterpenoids.

### 3.4. Validation of HPLC and TLC Methods

Prior to the establishment of the HPLC fingerprint, the precision, repeatability, and stability were chosen to validate the reliability of HPLC, which were expressed by the relative standard deviations (RSDs) of the retention time (*t*_*R*_) and peak area (Pa). For the precision test, the working solutions were analyzed in triplicate, and RSD values of *t*_*R*_ and Pa were lower than 0.2% and 4% (Table 2). To confirm the repeatability, five different working solutions prepared from the same batch of the sample were analyzed. The repeatability test (Table 2) demonstrated that the developed assay was reproducible (RSD < 5%). Stability of 11 analytes was detected at 2, 4, 8, 12, and 24 h, respectively. RSD values of Pa for stability tests were 1.46–4.81% (Table 2), which indicated that the sample was stable in 24 h.

In contrast with HPLC, TLC fingerprints were more cost-effective and provided a vivid colorful image for parallel comparison [20]. At present, there are few literatures about TLC fingerprints applied to *Ganoderma*. In this study, 10 batches of *Ganoderma* and the standard herb were further investigated by TLC fingerprints. In the preliminary experiment, developing solvent, sample concentration, chromogenic reagent, chromogenic temperature, and chromogenic time were optimized to achieve the optimum effect of separation and coloration. Owing to the large polarity of triterpenoids, a small amount of formic acid was added in the developing agent. Then, 1% vanillin-sulfuric acid solution was chosen as a chromogenic agent to color the compounds of triterpenoids. In order to validate the reliability of the TLC method, precision, repeatability, and stability were determined. The operation methods of working solutions were the same as HPLC. As shown in Table 3, RSDs of the flow rate value (*R*_*f*_) ranged from 0.33% to 2.48%, which proved that the TLC experiments were reliable. However, RSDs of the peak area value (Pa) of the main characteristic peaks were from 2.32% to 7.79% (Table 3), inferring that a certain amount of error was produced by manual spotting.

### 3.5. Establishment of Global Chemical Profile by ChemPattern Software

Both common patterns of HPLC and TLC fingerprints for triterpenoids were generated to represent the characteristic peaks of this authenticated herbal medicine by ChemPattern software (Chemmind Technologies, Beijing, China). The similarity and clustering analyses on different kinds of chromatographic fingerprint data comprehensively evaluated the types and quantities of triterpenoids by ChemPattern software.

**Figure 5:**
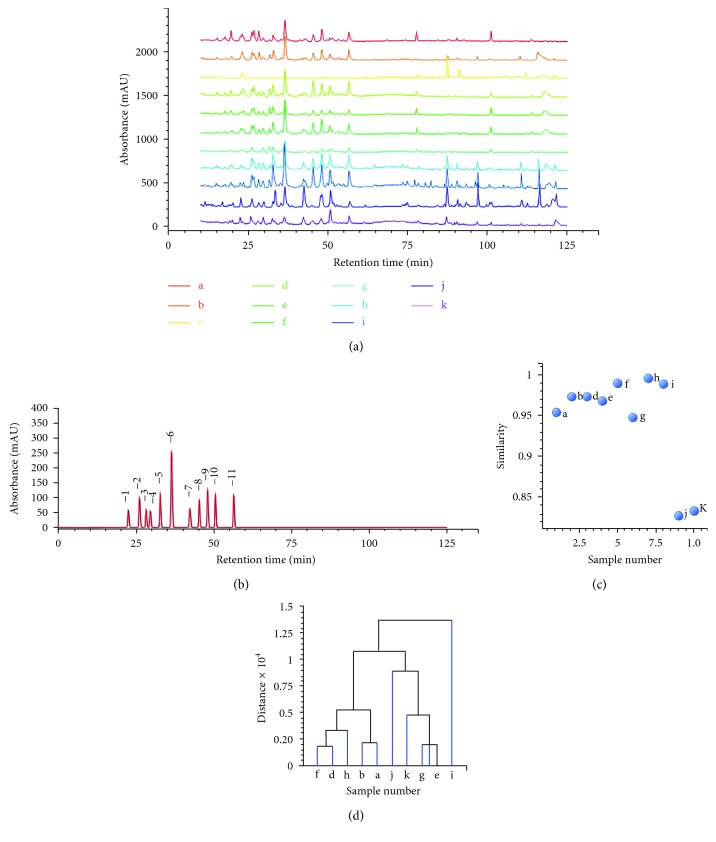
HPLC fingerprints of triterpenoids obtained from eleven batches of samples (a) and the common pattern generated by ChemPattern software (b). The similarity analysis (c) and clustering analysis (d) of triterpenoids.

The HPLC common pattern of 10 batches of samples is shown in Figures 5(a) and 5(b). Sample c from America was not suitable to establish the common model of the HPLC fingerprint because of its significant differences in chemical composition. 11 peaks existing in 10 batches of samples were found as common peaks. According to the compound database of triterpenoids obtained from HPLC-LTQ-Orbitrap-MS^*n*^, peaks 1 to 11 were identified as lucidenic acid LM_1_, ganoderic acid G, ganoderic acid B, lucidenic acid E, ganoderenic acid A, ganoderic acid A, lucidenic acid A, ganoderenic acid D, ganoderic acid D, lucidenic acid D, and ganoderic acid F, respectively. The relative retention time (*t*′_*R*_ = retention time of the characteristic peak/retention time of the marker peak) and relative peak area (RPA = peak area of the characteristic peak/peak area of the marker peak) of the common peaks are shown in Tables S4 and S5. Peak 6, identified as ganoderic acid A, was selected as the reference peak. The results indicated that *t*′_*R*_ of 11 common peaks (between 0.13% and 0.57%) was invariable between samples, and *t*′_*R*_ was a valid parameter for constituent identification. However, the RPA (from 29.19% to 135.20%) showed significant differences between 10 batches of samples, indicating that the content of triterpenoids from various sources was different.

The similarity and clustering analyses of HPLC fingerprints were also analyzed by ChemPattern software. The advanced chemical pattern recognition module of ChemPattern could forecast the classification of complex samples. The similarities of samples a, b, d, e, f, g, h, and i were greater than 0.830 (Figure 5(c)). However, the profiles of samples j and k were different from the common pattern, suggesting that these two samples belonged to *Ganoderma sinense*. This method could distinguish *Ganoderma sinense* and *Ganoderma lucidum* quickly. When the clustering distance was extended to 1.0, the samples from different regions could be divided into three groups. As shown in Figure 5(d), samples f, d, h, b, and a belonged to class I. These five samples were collected from Anhui Province. Samples k, j, g, and e were classified into class II, for the reason that the triterpenoid content and species of these samples were similar. Sample i was classified into class III individually, due to the different categories of triterpenoids. These results illustrated that the origins of *Ganoderma* could be distinguished via clustering analysis. The HPLC fingerprints could not only reflect the chemistry information of *Ganoderma* but also distinguish the *Ganoderma* species from different geographical origins.

**Figure 6:**
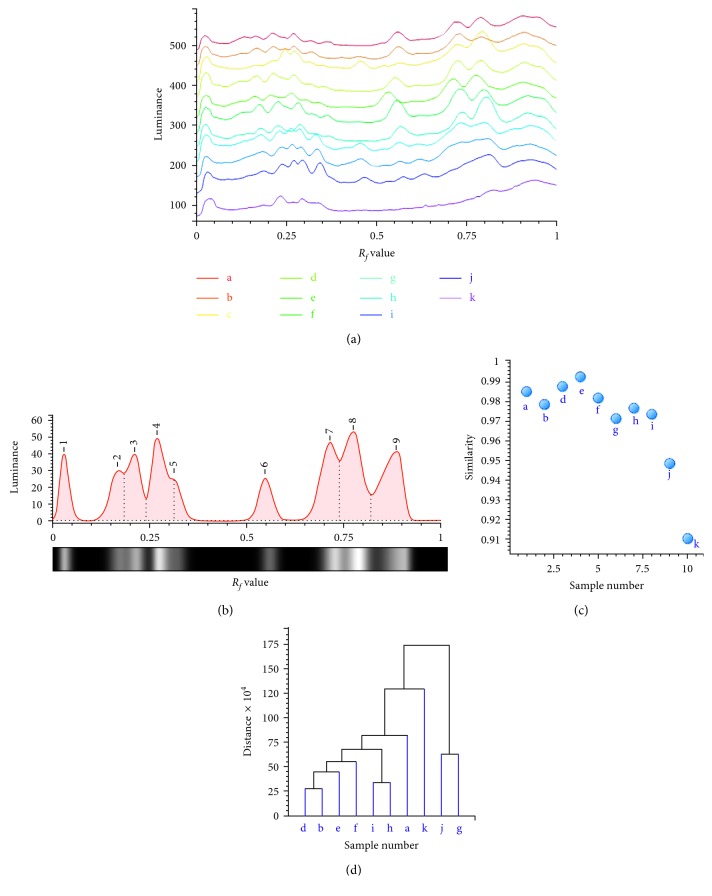
TLC fingerprints of triterpenoids obtained from different *Ganoderma* species (a) and their common pattern (b). The similarity analysis (c) and clustering analysis (d) of triterpenoids.

The image of TLC taken by a digital camera was reverse-phase processed before imported into ChemPattern software (Figure S2). Twelve tracks were set manually, and the information of TLC spots was turned into the gray curve. TLC fingerprints of different triterpenoid extracts and the common model were obtained as shown in Figures 6(a) and 6(b). The fingerprints exhibited that nine common peaks were likely to represent the major constituents of triterpenoids. The similarity and clustering analyses of TLC by ChemPattern software are shown in Figures 6(c) and 6(d). The similarities of the samples a, b, d, e, f, g, h, i, j, and k were all greater than 0.900, while samples j and k were relatively low, which was consistent with the result of the HPLC fingerprint (Figure 6(c)). The clustering analysis showed that the samples could be divided into three clusters if the Euclidean distance was equal to 25 (Figure 6(d)). The samples e, d, b, f, i, h, and a belonged to class I. Among these samples, only samples e and h were not collected from Anhui. Sample k was classified into class II individually, while samples j and g were classified into class III. The cluster analysis of 10 batches of *Ganoderma* showed a clear separation of the three groups, and common peaks played key roles in differentiating these samples. Both HPLC and TLC fingerprint classifications could provide a simple reference standard for quality identification of *Ganoderma*.

### 3.6. Discussion

In this article, RSM with the BBD method was successfully applied to optimize the factors for the ultrasonic extraction of triterpenoids. The suitable conditions were as follows: liquid-solid ratio 28 mL/g, ethanol concentration 87%, and extraction time 36 min at 50°C. The ultrasonic extraction technology has the advantage of accelerating the extraction time, lowering the temperature, causing less damage to the structure of plant materials, and increasing the extraction yield. It is more suitable for the extraction of triterpenoids. RSM is an effective statistical method useful for optimizing a complex process and evaluating the interaction between multiple parameters [21]. The yield of triterpenoids of 7.338 ± 0.150 mg/g could be attained, which was consistent with the theoretical predicted value (7.3118 mg/g). Therefore, the model was considered to be reliable, and RSM could be used for predicting the ultrasonic extraction yield of triterpenoids.

HPLC-LTQ-Orbitrap-MS^*n*^ technique was applied to identify the chemical structure of triterpenoids in *Ganoderma* with higher sensitivity. It provided high resolution and abundant structural information for not only the pseudomolecular ions but also the fragment species. The application of HPLC-LTQ-Orbitrap-MS^*n*^ could provide a large amount of information related to the chemical structure and make up the deficiency of UV and DAD detectors [8]. Based on the accurate mass measurement, MS/MS fragmentation patterns, and diagnostic product ions provided by HPLC-LTQ-Orbitrap-MS^*n*^ and literatures, 24 compounds were identified, which was one of the major tools for the study of the chemical substance of *Ganoderma*.

To investigate triterpenoids further, the chromatographic fingerprint method was applied to analyze the complex composition of *Ganoderma*. The fingerprint analysis technology is different from traditional analysis methods because it analyzes objects from the perspective of whole component information. The chromatographic fingerprint method was established through importing HPLC and TLC profiles into ChemPattern software for the comprehensive quality control of *Ganoderma*. The common pattern of fingerprints provided the critical information for constructing chemical fingerprints of triterpenoids. In addition, the fingerprint method combined with the stoichiometry could be applied to identify the species and origins of *Ganoderma*. The results obtained by clustering analysis of HPLC and TLC fingerprints were slightly different due to the fact that HPLC possesses high resolution and reproducibility. The correct classification percentages of the pattern recognition method of HPLC fingerprint identification were higher than those of TLC. However, in contrast with the HPLC fingerprint, the TLC fingerprint was more cost-effective and provided a vivid colorful image for parallel comparison. In summary, the chemical fingerprint method could be adopted as a reliable tool for the authentication and quality control of *Ganoderma*.

## 4. Conclusion

In the present study, the chemical composition of triterpenoids was clarified by HPLC-LTQ-Orbitrap-MS^*n*^, and the global chemical profile consisting of HPLC and TLC fingerprints was established. Eleven triterpenoid peaks which differed significantly in all the analyzed samples were used as markers for origin identification and authenticity establishment of *Ganoderma*. This work suggested that the developed global chemical profiling method could provide a convenient approach, which might be applied for rapid evaluation, quality control, and authenticity establishment of *Ganoderma* products. In the future, the chemical constituents and pharmacological activities of *Ganoderma* would be explored in depth through the multidimensional fingerprints combined with chemometric methods, molecular biology, and pattern recognition techniques.

## Figures and Tables

**Table 1:** Compounds identified in triterpenoids from *Ganoderma*.

Number	*t*_*R*_^a^ (min)	[M – H]^−^ (*m*/*z*)	MS/MS main fragment ions	Compound	Formula	Weight^b^	CAS number
1	33.88	517.3179	499.3361, 481.3423, 455.5135, 437.4728, 422.2257	Ganoderic acid C_2_	C_30_H_46_O_7_	518.68	103773-62-2
2	34.72	475.2600	457.33624, 439.5103	Lucidenic acid C	C_27_H_40_O_7_	476.60	95311-96-9
3	38.40	459.2756	441.3874, 423.3755	Lucidenic acid LM_1_	C_27_H_40_O_6_	460.60	364622-33-3
4	39.49	529.2814	481.2692, 467.3972, 437.4240, 407.4240	Ganoderic acid C_6_	C_30_H_42_O_8_	530.65	105742-76-5
5	43.11	531.2983	495.4055, 469.3644, 454.3719, 436.3896, 407.4450, 379.1423	Ganoderic acid G	C_30_H_44_O_8_	532.67	98665-22-6
6	43.69	513.2871	495.4676, 477.3961, 469.3892, 451.4328, 437.3875	Ganoderenic acid B	C_30_H_42_O_7_	514.65	100665-41-6
7	45.78	515.3024	479.3840, 453.3503, 438.3453, 409.3114, 391.4153	Ganoderic acid B	C_30_H_44_O_7_	516.67	81907-61-1
8	47.53	515.2659	497.3039, 473.3125, 455.1528, 410.9863	Lucidenic acid E	C_29_H_40_O_8_	516.62	98665-17-9
9	47.73	513.2869	495.4192, 469.4687, 451.3153, 436.2914	Ganoderic acid AM_1_	C_30_H_42_O_7_	514.65	149507-55-1
10	49.28	571.2925	553.4242, 538.6849, 511.4037	Ganoderic acid H	C_32_H_44_O_9_	572.69	98665-19-1
11	50.28	513.2868	498.3824, 495.3860, 469.3463, 439.3085, 424.2103, 406.3151	Ganoderenic acid A	C_30_H_42_O_7_	514.65	100665-40-5
12	51.28	527.2643	509.4469, 481.3122, 452.3277, 390.8892	Elfvingic acid A	C_30_H_40_O_8_	528.63	433284-49-2
13	53.29	473.2554	437.3959, 425.3581, 411.2774, 393.3146, 375.2134	Lucidenic acid B	C_27_H_38_O_7_	474.59	95311-95-8
14	54.09	515.4518	497.3640, 479.3914, 435.3200, 417.2299	Ganoderic acid A	C_30_H_44_O_7_	516.30	81907-62-2
15	55.28	499.3073	481.4785, 479.3914, 437.4701, 419.3591	Ganolucidic acid A	C_30_H_44_O_6_	500.67	98665-21-5
16	59.28	457.2603	442.3622, 439.2677, 421.2575, 413.4738, 395.4018	Lucidenic acid A	C_27_H_38_O_6_	458.59	95311-94-7
17	60.57	455.2449	437.3401, 425.2315, 411.5940, 393.3439, 383.2032, 365.0397	Lucidenic acid F	C_27_H_36_O_6_	456.57	98665-18-0
18	61.32	513.2871	478.2901, 463.2901, 449.2825, 434.4029	Ganoderenic acid D	C_30_H_40_O_7_	512.63	100665-43-8
19	63.77	495.2764	477.3036, 451.3039, 436.3244, 407.4024, 365.2903	Ganoderic acid D	C_30_H_42_O_7_	514.65	108340-60-9
20	65.45	513.2506	495.3201, 471.2714, 453.1822, 425.5560, 396.4717	Lucidenic acid D	C_29_H_38_O_8_	514.61	98665-16-8
21	66.29	511.2711	493.4020, 467.4334, 449.3544, 434.2898	Ganoderic acid E	C_30_H_40_O_7_	512.27	98665-14-6
22	67.26	499.3074	481.4776, 437.3365, 419.4566	Ganolucidic acid D	C_30_H_44_O_6_	500.67	102607-22-7
23	69.07	569.2768	521.3403, 509.2697, 465.3616, 447.4046	Ganoderic acid F	C_32_H_42_O9	570.67	98665-15-7
24	70.18	513.2867	495.3300, 471.3816, 451.3528, 436.4189, 421.2986	Ganoderic acid J	C_30_H_42_O_7_	514.65	100440-26-4

^a^Retention time; ^b^relative molecular weight.

**Table 2:** Precision, repeatability, and stability of eleven analytes in HPLC.

Analyte	Mean of *t*_*R*_^a^	Precision (*n* = 3)		Repeatability (*n* = 5)		Stability (*n* = 5)						
		RSD (%) of *t*_*R*_	RSD (%) of Pa^b^	RSD (%) of *t*_*R*_	RSD (%) of Pa	RSD (%) of *t*_*R*_	Pa					RSD (%) of Pa
							2 h	4 h	8 h	12 h	24 h	
1	22.13	0.15	2.22	0.05	3.02	0.14	992	1004	1018	1054	1074	3.34
2	25.66	0.19	3.40	0.05	4.90	0.21	2022	2193	2118	2092	2277	4.57
3	27.74	0.13	3.33	0.05	3.98	0.14	2191	2124	2147	2132	2268	2.73
4	29.02	0.08	1.14	0.06	3.89	0.13	2209	2059	2192	2297	2333	4.81
5	32.15	0.09	3.37	0.05	2.85	0.06	4570	4687	4851	4783	4817	2.40
6	35.78	0.11	0.44	0.05	3.93	0.07	9187	9299	9480	9510	9681	1.88
7	41.51	0.07	2.96	0.06	4.63	0.16	2040	2128	2090	2283	2193	4.40
8	44.57	0.04	2.44	0.03	2.98	0.11	3941	4003	3984	4056	3905	1.46
9	47.32	0.02	0.77	0.02	2.68	0.07	4703	4923	4957	4924	4993	2.32
10	49.67	0.12	3.02	0.05	2.89	0.19	3919	3822	3913	3723	3827	2.09
11	55.67	0.10	3.87	0.03	2.68	0.13	3656	3803	3748	3756	3861	1.92

^a^Retention time of the analytes; ^b^peak area of the analytes.

**Table 3:** Precision, repeatability, and stability of nine analytes in TLC.

Analyte	Mean of *R*_*f*_^a^	Precision (*n* = 3)		Repeatability (*n* = 5)		Stability (*n* = 5)						
		RSD (%) of *R*_*f*_	RSD (%) of Pa^b^	RSD (%) of *R*_*f*_	RSD (%) of Pa	RSD (%) of *R*_*f*_	Pa					RSD (%) of Pa
							2 h	4 h	8 h	12 h	24 h	
1	0.03	0.75	6.81	0.54	4.88	0.86	72.88	75.74	73.45	79.74	72.44	4.03
2	0.19	0.40	3.69	1.61	3.93	2.09	68.49	59.77	66.73	70.52	69.87	6.46
3	0.23	0.87	3.74	1.05	5.44	0.56	88.74	84.91	90.11	85.77	90.45	2.87
4	0.27	1.52	6.29	1.34	3.74	2.48	121.01	125.74	128.44	131.34	130.22	3.24
5	0.33	1.01	6.60	1.01	3.81	1.11	64.41	64.88	66.86	70.11	70.29	4.15
6	0.54	0.37	4.76	0.84	7.64	0.52	31.22	28.87	30.21	33.47	34.49	7.30
7	0.72	1.11	6.10	1.03	7.79	0.39	155.70	149.38	145.06	156.63	149.74	3.18
8	0.80	0.68	7.33	1.43	3.14	1.07	135.75	133.96	141.01	142.13	139.54	2.52
9	0.90	0.94	4.18	0.33	3.78	0.97	117.25	131.49	134.84	129.71	128.56	2.32

^a^ *R*_*f*_ value of the analytes; ^b^peak area of the analytes.

## Supplementary Materials

Supplementary Materials Table S1: Factors and levels in extraction experiments. Table S2: Box-Behnken design of extraction parameters. Table S3: ANOVA for the response surface quadratic model of extraction parameters. Table S4: The *t*′_*R*_^a^ of the common peak in different *Ganoderma* species. Table S5: The RPA^b^ of the common peak in different *Ganoderma* species. Figure S1: The structures of compounds identified in triterpenoids from *Ganoderma*. Figure S2: TLC image of triterpenoids (a) and the reverse-phase processing image (b). Twelve tracks from left to right were ganoderic acid A and samples a, b, c, d, e, f, g, h, i, j, and k, respectively.
