# Supplementary material for: Development of Global Chemical Profiling for Quality Assessment of Ganoderma Species by ChemPattern Software
Source: J Anal Methods Chem. 2018 Mar 4;2018:1675721. doi: 10.1155/2018/1675721 (PMC5857333; doi:10.1155/2018/1675721)
Supplement: Supplementary Materials — Table S1: Factors and levels in extraction experiments. Table S2: Box-Behnken design of extraction parameters. Table S3: ANOVA for the response surface quadratic model of extraction parameters. Table S4: The t′R a of the common peak in different Ganoderma species. Table S5: The RPAb of the common peak in different Ganoderma species. Figure S1: The structures of compounds identified in triterpenoids from Ganoderma. Figure S2: TLC image of triterpenoids (a) and the reverse-phase processing image (b). Twelve tracks from left to right were ganoderic acid A and samples a, b, c, d, e, f, g, h, i, j, and k, respectively. [file 1675721.f1.doc]

**Supporting Information to:**

**Development of Global Chemical Profiling for Quality Assessment of *Ganoderma* Species by ChemPattern Software**

Hui Zhang, Huijie Jiang, Xiaojing Zhang, Shengqiang Tong, Jizhong Yan[[1]](#footnote-2)

TABLE S1. Factors and levels in extraction experiments.

| Factors | Levels | | |
| --- | --- | --- | --- |
|  | -1 | 0 | 1 |
| Liquid-solid ratio (A) / (mL/g) | 15 | 25 | 35 |
| Extraction time (B) / (min) | 30 | 45 | 60 |
| Ethanol concentration (C) / (%) | 75 | 85 | 95 |

TABLE S2. Box-Behnken design of extraction parameters.

| No. | A | B | C | Y |
| --- | --- | --- | --- | --- |
| 1 | -1 | -1 | 0 | 7.062 |
| 2 | 1 | -1 | 0 | 7.115 |
| 3 | -1 | 1 | 0 | 7.073 |
| 4 | 1 | 1 | 0 | 6.924 |
| 5 | -1 | 0 | -1 | 6.074 |
| 6 | 1 | 0 | -1 | 6.157 |
| 7 | -1 | 0 | 1 | 6.701 |
| 8 | 1 | 0 | 1 | 6.834 |
| 9 | 0 | -1 | -1 | 6.298 |
| 10 | 0 | 1 | -1 | 5.868 |
| 11 | 0 | -1 | 1 | 6.926 |
| 12 | 0 | 1 | 1 | 6.513 |
| 13 | 0 | 0 | 0 | 7.196 |
| 14 | 0 | 0 | 0 | 7.307 |
| 15 | 0 | 0 | 0 | 7.289 |
| 16 | 0 | 0 | 0 | 7.152 |
| 17 | 0 | 0 | 0 | 7.221 |

TABLE S3. ANOVA for response surface quadratic model of extraction parameters.

| Source | Sum of  Squares | df | Mean  Square | *F* Value | *P*-value  Prob > *F* |  |
| --- | --- | --- | --- | --- | --- | --- |
| Model | 3.298 | 9 | 0.366 | 30.56 | < 0.0001 | significant |
| A | 0.002 | 1 | 0.002 | 0.15 | 0.7099 |  |
| B | 0.131 | 1 | 0.131 | 10.91 | 0.0131 |  |
| C | 0.830 | 1 | 0.830 | 69.24 | < 0.0001 |  |
| AB | 0.010 | 1 | 0.010 | 0.85 | 0.3870 |  |
| AC | 0.001 | 1 | 0.001 | 0.05 | 0.8259 |  |
| BC | 0.000 | 1 | 0.000 | 0.01 | 0.9403 |  |
| A^2^ | 0.023 | 1 | 0.023 | 1.96 | 0.2047 |  |
| B^2^ | 0.056 | 1 | 0.056 | 4.63 | 0.0683 |  |
| C^2^ | 2.164 | 1 | 2.164 | 180.49 | < 0.0001 |  |
| Residual | 0.084 | 7 | 0.012 |  |  |  |
| Lack of Fit | 0.067 | 3 | 0.022 | 5.37 | 0.0690 | not significant |
| Pure Error | 0.017 | 4 | 0.004 |  |  |  |
| Cor Total | 3.382 | 16 |  |  |  |  |

*P* < 0.05 means significant; *P* < 0.01 means highly significant.

TABLE S4. The t_R_’ ^a)^ of common peak in different *Ganoderma*.

| No. | The t_R_’ of common peak | | | | | | | | | | | |
| --- | --- | --- | --- | --- | --- | --- | --- | --- | --- | --- | --- | --- |
|  | | 1 | 2 | 3 | 4 | 5 | 6 | 7 | 8 | 9 | 10 | 11 |
| a | | 0.616 | 0.714 | 0.774 | 0.812 | 0.900 | 1.000 | 1.163 | 1.247 | 1.321 | 1.390 | 1.554 |
| b | | 0.628 | 0.716 | 0.775 | 0.813 | 0.900 | 1.000 | 1.161 | 1.244 | 1.319 | 1.387 | 1.550 |
| d | | 0.617 | 0.716 | 0.775 | 0.813 | 0.900 | 1.000 | 1.162 | 1.246 | 1.320 | 1.389 | 1.552 |
| e | | 0.616 | 0.715 | 0.774 | 0.813 | 0.898 | 1.000 | 1.161 | 1.245 | 1.320 | 1.389 | 1.552 |
| f | | 0.618 | 0.715 | 0.775 | 0.814 | 0.900 | 1.000 | 1.162 | 1.246 | 1.321 | 1.390 | 1.555 |
| g | | 0.617 | 0.713 | 0.774 | 0.813 | 0.898 | 1.000 | 1.161 | 1.244 | 1.320 | 1.389 | 1.553 |
| h | | 0.619 | 0.716 | 0.775 | 0.815 | 0.899 | 1.000 | 1.163 | 1.247 | 1.322 | 1.391 | 1.555 |
| i | | 0.617 | 0.716 | 0.775 | 0.813 | 0.900 | 1.000 | 1.166 | 1.250 | 1.325 | 1.396 | 1.561 |
| j | | 0.619 | 0.715 | 0.773 | 0.818 | 0.898 | 1.000 | 1.166 | 1.242 | 1.323 | 1.398 | 1.562 |
| k | | 0.616 | 0.710 | 0.770 | 0.817 | 0.895 | 1.000 | 1.166 | 1.249 | 1.322 | 1.400 | 1.564 |
| Mean | | 0.618 | 0.715 | 0.774 | 0.842 | 0.899 | 1.000 | 1.163 | 1.246 | 1.321 | 1.392 | 1.556 |
| RSD（%） | | 0.57 | 0.25 | 0.19 | 0.23 | 0.17 | 0.00 | 0.19 | 0.19 | 0.13 | 0.32 | 0.30 |

1. t_R_’ = retention time of common peak / retention time of marker peak)

TABLE S5. The RPA ^b)^ of common peak in different *Ganoderma*.

| No. | | The RPA of common peak | | | | | | | | | | | | |
| --- | --- | --- | --- | --- | --- | --- | --- | --- | --- | --- | --- | --- | --- | --- |
|  | 1 | | 2 | | 3 | 4 | 5 | 6 | 7 | 8 | | 9 | 10 | 11 |
| a | 0.231 | | 0.337 | | 0.461 | 0.140 | 0.290 | 1.000 | 0.074 | | 0.343 | 0.569 | 0.119 | 0.378 |
| b | 0.337 | | | 0.316 | 0.270 | 0.147 | 0.378 | 1.000 | 0.076 | | 0.293 | 0.421 | 0.172 | 0.332 |
| d | 0.125 | | | 0.261 | 0.155 | 0.155 | 0.482 | 1.000 | 0.096 | | 0.544 | 0.384 | 0.273 | 0.457 |
| e | 0.222 | | | 0.372 | 0.203 | 0.200 | 0.472 | 1.000 | 0.076 | | 0.272 | 0.254 | 0.216 | 0.245 |
| f | 0.173 | | | 0.270 | 0.145 | 0.202 | 0.388 | 1.000 | 0.143 | | 0.409 | 0.471 | 0.241 | 0.329 |
| g | 0.170 | | | 0.356 | 0.139 | 0.392 | 0.355 | 1.000 | 0.087 | | 0.115 | 0.229 | 0.224 | 0.302 |
| h | 0.098 | | | 2.780 | 0.187 | 0.205 | 0.428 | 1.000 | 0.231 | | 0.357 | 0.512 | 0.404 | 0.402 |
| i | 0.084 | | | 0.252 | 0.153 | 0.159 | 0.449 | 1.000 | 0.157 | | 0.436 | 0.502 | 0.339 | 0.341 |
| j | 0.312 | | | 0.337 | 0.194 | 0.223 | 0.221 | 1.000 | 1.179 | | 0.036 | 0.514 | 0.813 | 0.263 |
| k | 0.677 | | | 0.942 | 0.467 | 0.784 | 0.666 | 1.000 | 1.117 | | 0.356 | 0.886 | 1.875 | 0.893 |
| Mean | 0.243 | | | 0.622 | 0.237 | 0.261 | 0.413 | 1.000 | 0.324 | | 0.316 | 0.474 | 0.468 | 0.394 |
| RSD（%） | 71.68 | | | 126.07 | 52.78 | 75.89 | 29.19 | 0.00 | 135.20 | | 47.23 | 38.63 | 113.61 | 47.23 |

1. RPA= peak area of common peak / peak area of marker peak

FIGURE S1. The structures of compounds identified in triterpenoids from *Ganoderma*.


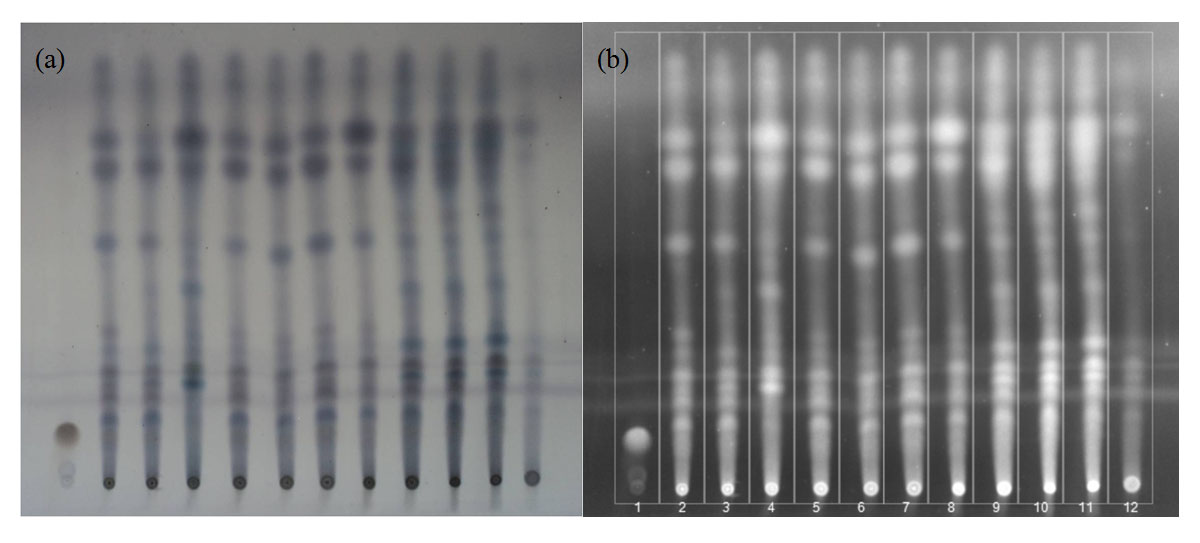


FIGURE S2. TLC image of triterpenoids (a) and the reversed-phase processing image (b). Twelve tracks from left to right were ganoderic acid A, sample a, b, c, d, e, f, g, h, i, j and k, respectively.

1. Corresponding to: Jizhong Yan, College of Pharmaceutical Science, Zhejiang University of Technology, Hangzhou 310014, China.

   E-mail: [science5555@163.com](mailto:science5555@163.com)

   College of Pharmaceutical Science, Zhejiang University of Technology, Hangzhou 310014, China. [↑](#footnote-ref-2)
